# Effect of Group Impromptu Music Therapy on Emotional Regulation and Depressive Symptoms of College Students: A Randomized Controlled Study

**DOI:** 10.3389/fpsyg.2022.851526

**Published:** 2022-03-31

**Authors:** Ming Zhang, Yi Ding, Jing Zhang, Xuefeng Jiang, Nannan Xu, Lei Zhang, Wenjie Yu

**Affiliations:** ^1^Qingdao Medical College, Qingdao University, Qingdao, China; ^2^Business School of Qingdao University, Qingdao, China; ^3^The Affiliated Hospital of Qingdao University, Institute for Translational Medicine, Qingdao University, Qingdao, China

**Keywords:** college students, group impromptu music therapy, emotional regulation, depressive symptom, improvement

## Abstract

Difficulty in emotional regulation is significantly correlated with depression. Depression is a psychological disease that seriously affects the physical and mental health of college students. Therefore, it is of great importance to develop diversified preventive interventions such as group impromptu music therapy (GIMT). The main purpose of this study was to evaluate the effect of GIMT on the improvement of emotional regulation ability and the reduction of depressive symptoms in college students. A 71 college students (36 in the experimental group and 35 in the control group) were recruited to carry out randomized controlled trial was used. The experimental group was intervened by GIMT. After the 4th week of intervention, follow-up and scale measurement were carried out. In the experimental group, emotional regulation difficulty scales (DERS) showed significant difference before and after GIMT, implying the improvement in the emotional regulation. But there was no significant improvement in the control group. In addition, the depressive symptoms of experimental group were relieved. All findings showed that GIMT can effectively improve college students’ emotional regulation and reduce depressive symptoms.

## Introduction

Emotional regulation refers to the processes of modifying emotions, including how and when emotions are experienced and expressed (Gross, 2002; Nolen-Hoeksema, 2012). Studies have shown that emotional regulation ability is significantly correlated with individual social function, emotional state, cognitive level, and academic development. The difficulty of emotional regulation will make the individual’s subjective emotional experience worse and gradually expand their negative emotions, aggravating the symptoms of depression.

As the most common mental disease in the world (Vos et al., 2015), depression is regarded as an emotional regulation disorder (Compare et al., 2014). Difficulty in emotional regulation is the core symptom of major depression and an important risk factor for the development of depression (Berking et al., 2014). Moreover, difficulty in emotional regulation plays a negative role in the development and deterioration of depression (Joormann and Quinn, 2014). Generally, people suffering from depression might encounter difficulties in negative emotional regulation (Herwig et al., 2018) and show symptoms of reduced positive emotions (Joormann and Stanton, 2016). In China, the incidence of depression is 26.57%, showing an upward trend year by year. Depression has become the main emotional problem of college students. In their early adulthood, college students are susceptible to emotional regulation difficulties and depression. Studies have found that the incidence of depression was higher among young people aged 18–25 (Breedvelt et al., 2018; Barker et al., 2019).

Generally, the application of adaptive coping strategies is an important premise for individuals to effectively implement emotional regulation (Rotter et al., 1972; Folkman and Moskowitz, 2004). Studies have proved that positive strategies such as seeking social support, relieving stress, and enhancing physical and mental health can effectively improve emotional regulation (Holahan and Moos, 1986; Surmann, 1999). To improve, the ability of emotional regulation and prevent depression is important for the physical and mental health development of college students (Ibrahim et al., 2013; Macdonald and Price, 2018; Aalbers et al., 2019). At present, the prevention and intervention for college students’ emotional disorders are mostly based on cognitive therapy (Conley et al., 2017; Breedvelt et al., 2018). However, some students, especially those experiencing emotional regulation pressure, may have difficulty in expressing their thoughts and feelings in language. So diversified prevention and intervention measures need to be developed, such as music therapy (Aalbers et al., 2019).

Music therapy is a method of psychotherapy, which stimulates and hypnotizes people in various forms of music activities, and stimulates physical response with sound, so as to improve mental health. The methods of music therapy mainly include reception, improvisation, creation, and recreation. Treatment methods can be divided into individual treatment and group treatment. A large number of studies have proved that music therapy, including impromptu music therapy, can effectively reduce depressive symptoms and improve physical and mental function (Erkkila et al., 2008, 2011; Aalbers et al., 2017). Within the framework of safe and standardized treatment, active improvisation and music creation can provide conditions for physical and mental experience based on esthetic (Maratos et al., 2011). Impromptu music therapy does not require patients to have music skills. So it is suitable for almost all groups and can be successfully applied even in the case of limited language ability (Jaakko, 2004; Backer, 2008). Adults with normal cognitive and abstract thinking abilities can associate the psychological content based on life experience with music expression in improvisation. And they can interpret or process these experiences according to the current situation (Erkkila et al., 2019). Based on extensive practical research, many music therapists believe that music therapy has potential positive significance for emotional regulation (Marik and Stegemann, 2016).

Group improvisational music therapy (GIMT) is one method of music therapy. Through collective improvisation of musical instruments, music therapists help the participants to vent negative emotions, establish interpersonal links, express themselves, and accept others in safe and nonverbal “quasi socialized” activities. This will improve emotional regulation and psychological adaptability. In GIMT, music therapists follow the principles of music synchronization and empathy, and refer to the “three-dimensional dynamic relationship.” Empathy, mirroring, dialogue, emotional exploration, and discussion are used to promote participants to achieve positive change in the interaction of impromptu music (Wigram, 2004).

Although it has long been known that music affects emotional experience and emotional expression (Blood and Zatorre, 2001; Koelsch et al., 2013a,b), there are few studies on the relationship between music and emotional regulation (Hou et al., 2017). The application of impromptu music therapy in emotional regulation of college students is even less. Aalbers et al. developed improvised music therapy (EIMT) in 2019. Their study proves that EIMT can effectively reduce the depressive symptoms of young students (Aalbers et al., 2019) and improve the ability of emotional regulation. However, EIMT is mainly developed for one-to-one individual therapy. The effectiveness verification of group impromptu music therapy (GIMT) technology is still blank.

In this paper, we applied GIMT to improve college students’ emotional regulation and depressive symptoms, which is the first time in this field. We adopted a randomized controlled trial design. Statistical methods were used to quantify the degree of students’ emotional changes. Semi-structured interviews were used to qualitatively analyze the intervention effect (Fetters et al., 2013; Fetters and Molina-Azorin, 2019). We combined quantitative and qualitative results to comprehensively evaluate the effect of GIMT on depression and emotional regulation.

## Materials and Methods

### Subjects

From October 2019 to July 2020, the project team conducted recruitment and test. The subjects were students (478) from a university in Qingdao, China. The selection methods and criteria are as: (a) undergraduate students; (b) between 18 and 20 years of age; and (c) all subjects were collectively tested with the Difficulties in Emotion Regulation Scale (DERS) and Beck Depression Inventory (BDI). The exclusion criteria are as: (a) having urgent suicidal thoughts or behaviors; (b) past or present mental illness; and (c) receiving music therapy or antidepressant treatment elsewhere at present. A 71 students with DERS ≥ 101, BDI score ≥ 10, and depressive symptoms were finally included in the experiment. They were randomly divided into two groups, including 36 in the experimental group (16 males and 20 females) and 35 in the control group (18 males and 17 females). The experimental group signed informed consent and confidentiality agreement. The whole protocol of this study was admitted by the ethics committee of the Affiliated Hospital of Qingdao University.

### Scales

DERS is one of the important means of measuring emotional regulation. DERS is a 5-point Likert scale with 36 items, testing six emotional dimensions: emotional perception, emotional understanding, emotional response acceptance, emotional impulse control, goal-directed behavior stimulation, and effective use of emotion regulation strategies (Gratz and Roemer, 2004). The DERS score is negatively correlated with the degree of improvement. The higher the score, the worse the ability of emotional regulation (Gratz and Roemer, 2004).

BDI is one of the most widely used scales to measure depressive symptoms under multicultural background (Boyd et al., 2005; Manian et al., 2013; Wu and Huang, 2014). The higher the total score, the more serious the depression. The grading criteria are as: BDI < 10, no depressive symptoms; BDI ≥ 10, suspected depression; and BDI ≥ 15: depression. The BDI scale has good stability (test–retest reliability) among Chinese college students.

### GIMT Intervention

The intervention implementers are certified and registered music therapists. The assistants are two certified psychological counselors from the school mental health education center. The project director is a certified and registered music therapist who has received GIMT system training. The director did not participate in GIMT intervention to prevent bias. All of them have had more than 7 years of psychological counseling experience.

Under the guidance and promotion of music therapists, GIMT participants freely play percussion instruments with low technical threshold such as drums and jointly create personalized music belonging to individuals and the team. During the performance, the therapists follow the students’ performance synchronously to stimulate and amplify the students’ emotional experience and emotional resonance at any time. After each impromptu performance, the therapists will discuss with the students the emotional experience triggered by the performance.

GIMT is implemented in three stages. The first stage is to evaluate the emotional regulation ability and depression status, formulate plans, and determine the overall goal and the sub goals of the intervention. The second stage is the implementation of the plan. The third stage is to encourage students to use healthy emotional regulation strategies in their daily life and complete the assessment. In order to enhance the effectiveness, homework will be assigned after each intervention. Students are required to record their positive coping strategies and feelings in the face of emotional difficulties. Homework assignment is not common in music therapy, which is different from the usual cognitive therapy (Mausbach et al., 2010).

### Data Collection

The test project and data were collected from March 2020 to June 2020 in Qingdao University. Specific recruitment methods are as: (a) based on the freshmen’s mental health assessment carried out by the University, students with University Personality Inventory (UPI) ≥ 20 (such students may have some degree of psychological problems or are very likely to have psychological problems) are recruited; (b) For students reporting depressive symptoms, relevant interventions, including GIMT, are recommended; and (c) Recruitment through the college platform and poster board. All students join voluntarily.

DERS and BDI were measured in the designated classroom. Students with total scores of DERS ≥101 and BDI ≥ 10 were interviewed one by one. A 71 students with depressive symptoms were included in the experiment. After completing GIMT for 1 week, the students in the experimental group and the control group were collectively tested with DERS and BDI. Then, the two groups of students were followed up for 4 weeks. In particular, as a compensation, GIMT intervention with no follow-up evaluation was also performed for the control group after all tests.

### Experimental Scheme

**Figure 1:**
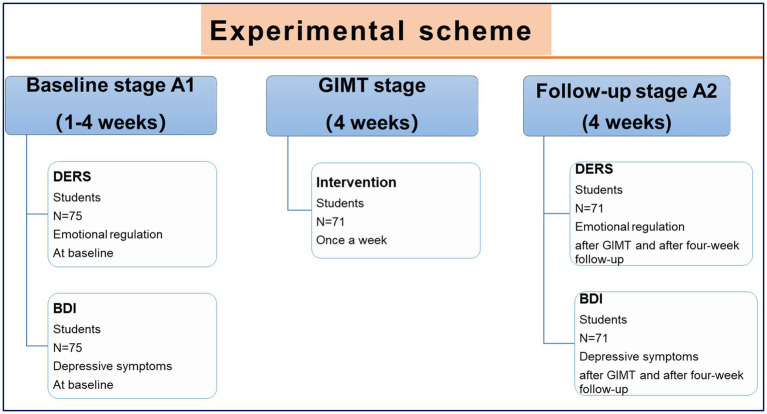
The data collection procedure. GIMT was applied to improve college students’ emotional regulation and depressive symptoms. The specific scheme was depicted in the figure.

The data collection procedure is shown below (Figure 1). Semi-structured interviews were used to collect information. The schedule was as follows: (A1) baseline-stage (4 weeks before pre-test): completing subject recruitment and screening; informed consent and random grouping; (B) GIMT intervention stage: lasted for 4 weeks, once a week; and (A2) post-test and follow-up stage (4 weeks): follow-up of subjects in non-intervention stage.

### Data Processing

The data distribution of each scale was analyzed by Graphpad prism software, and the differences between different groups were analyzed by Mann Whitney U test. All tests were bilateral or two tailed. The difference of *p* < 0.05 was statistically significant. The data were as: mean ± standard error.

## Results

### Characterization of Experimental and Control Subjects

**Figure 2:**
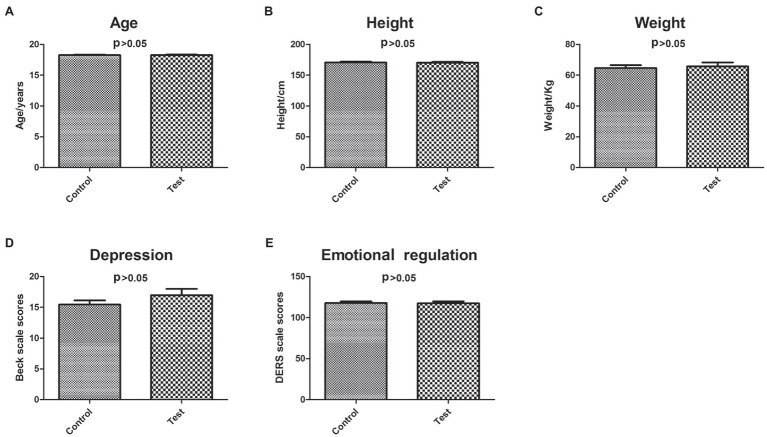
Characterization of control and experimental subjects. The differences between control and experimental subjects in age **(A)**, height **(B)**, weight **(C)**, depression **(D)**, and emotional regulation **(E)**.

There were no significant differences in age (18.27 ± 0.45 cm, 18.26 ± 0.56 cm, *p* = 0.9678), height (170.5 ± 8.3 cm, 170.2 ± 7.98, *p* = 0.9213), and weight (64.63 ± 10.31 kg, 65.71 ± 15.42 kg, *p* = 0.6780) between control and experimental groups (Figures 2A–C).

Before GIMT intervention, there were no significant differences in BDI (control group: 15.47 ± 3.64; experimental group: 16.97 ± 6.04, *p* = 0.4556) and DERS (control group: 117.69 ± 11.05; experimental group: 117.43 ± 14.32, *p* = 0.7420) between control and experimental groups (Figures 2D,E).

Before intervention, the overall emotional regulation levels of the two groups were similar. Among them, no significant differences were found in the dimensions of emotional understanding, emotional response acceptance, emotional impulse control, goal-directed behavior stimulation, and effective use of emotion regulation strategies. But there is significant difference in the dimension of emotional perception. The data of each dimension of DERS were shown in Table 1.

**Table 1:** Characterization of experimental and control subjects.

Categories	Control group	Experimental group	Value of *p*
Emotional regulation	117.69 ± 11.05	117.43 ± 14.32	0.7420
Emotional perception	18.6 ± 3.11	22.71 ± 4.18	<0.0001^***^
Emotional response acceptance	20.27 ± 3.26	19.26 ± 4.72	0.3761
Emotional understanding	15.83 ± 2.17	16.46 ± 3.19	0.5987
Goal-directed behavior stimulation	17.67 ± 2.25	17.51 ± 3.09	0.8946
Effective use of regulatory strategies	26.23 ± 3.78	24.18 ± 4.51	0.0506
Emotional impulse control	19.27 ± 2.26	18.21 ± 3.18	0.0529

*All data were obtained from the corresponding test scales and expressed as mean ± standard error.*

*** *p < 0.001*.

### Analysis of the Effect of GIMT Intervention on Depression

**Figure 3:**
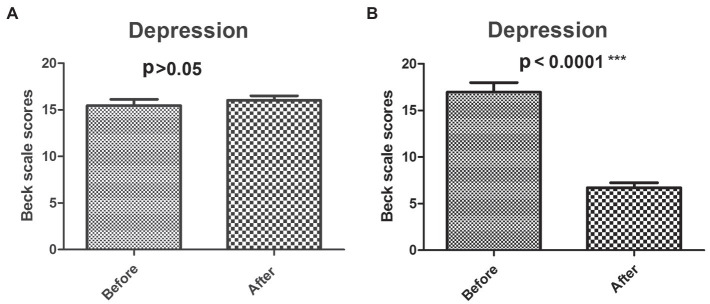
Analysis of BDI scores before and after intervention. The dynamic BDI scores before and after GMIT intervention in control group **(A)** and experimental group **(B)**. ^***^p < 0.001.

The differences of BDI scores before and after intervention between the experimental group and the control group were shown in Figure 3. In the control group with no intervention, there was no significant change in the depression level (before-test 15.47 ± 3.64, after-test 16.03 ± 2.57, *p* = 0.6993 > 0.05; Figure 3A). After intervention, depression level of the experimental group was significantly reduced (before-test 16.97 ± 6.04, after-test 6.71 ± 3.08, *p* < 0.0001; Figure 3B).

Considering gender factors, we classified the GIMT intervention results of boys and girls (Table 2). In the control group, no significant changes in the BDI scales in both girls and boys. In the experimental group, the improvement of depression in girls was significantly higher than that in boys.

**Table 2:** Gender differences of GIMT effect in two groups in depression.

Depression	Gender	Before	After	*p*
Control group	Male	16.13 ± 3.56	15.75 ± 2.11	0.7186
	Female	14.71 ± 3.71	16.36 ± 3.05	0.2681
Experimental group	Male	14.6 ± 5.18	6.92 ± 2.02	0.0002^**^
	Female	18.75 ± 6.15	7.12 ± 2.63	<0.0001^***^

*All data were obtained from the corresponding test scales and expressed as mean ± standard error*.

** *p < 0.01 and*

*** *p < 0.001*.

### Analysis of the Effect of GIMT Intervention on Emotional Regulation

**Figure 4:**
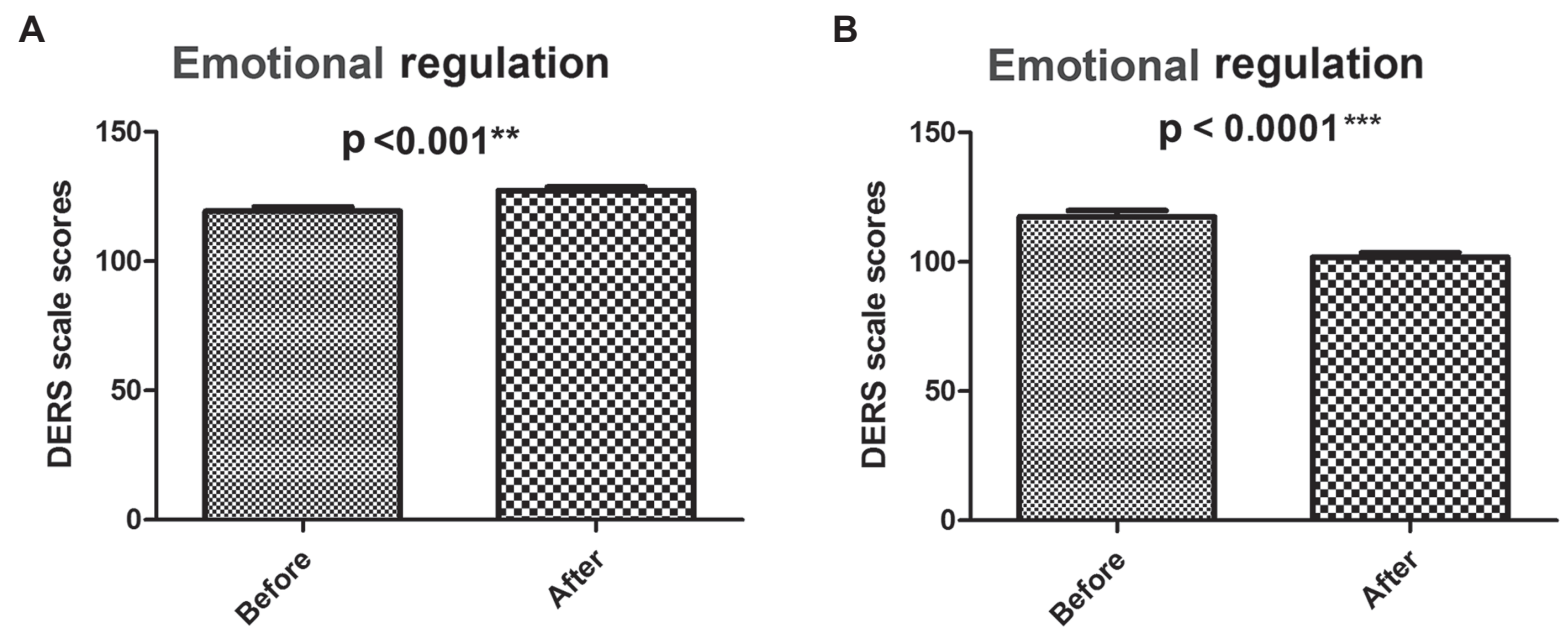
Analysis of total DERS scores before and after intervention. The dynamic DERS scores before and after GMIT intervention in control group **(A)** and experimental group **(B)**. ^**^p < 0.01 and ^***^p < 0.001.

The emotional regulation of both the experimental group and the control group had significant changes before and after GIMT intervention (Figures 4A,B). The DERS score of the control group was increased (before-test 117.69 ± 11.05, after-test 127.3 ± 7.78, *p* < 0.001^**^; Figure 4A), whereas the DERS score of the experimental group was reduced (before-test 117.43 ± 14.32, after-test 101.85 ± 9.79, *p* < 0.0001^**^; Figure 4B).

For control subjects with no GIMT intervention, there were no significant differences in the scores of emotional response acceptance, emotional understanding, and goal-directed behavior stimulation increased (Table 3), showing no improvement. However, in the two dimensions of effective use of regulatory strategies and emotional impulse control, the scores were significantly increased (Table 3), even showing a trend of deterioration.

**Table 3:** Dimension data of DERS scale of the control group before and after the intervention.

Control group	Before	After	Value of *p*
Emotional regulation	117.69 ± 11.05	127.3 ± 7.78	0.0008^**^
Emotional perception	18.6 ± 3.11	16.73 ± 2.45	0.0097^*^
Emotional response acceptance	20.27 ± 3.26	21.13 ± 2.73	0.1285
Emotional understanding	15.83 ± 2.17	16.1 ± 1.99	0.6420
Goal-directed behavior stimulation	17.67 ± 2.25	17.73 ± 2.02	0.9880
Effective use of regulatory strategies	26.23 ± 3.78	28.27 ± 3.03	0.0341^*^
Emotional impulse control	19.27 ± 2.26	21.2 ± 2.11	0.0014^*^

*All data were obtained from the corresponding test scales and expressed as mean ± standard error.*

* *p < 0.05 and*

** *p < 0.01*.

**Figure 5:**
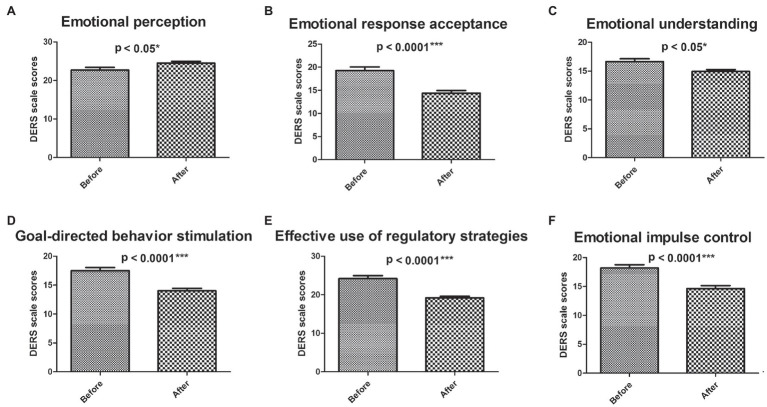
Analysis of DERS scores in six dimensions before and after intervention for experimental group. The dynamic DERS scores of six dimensions before and after GMIT intervention in control group and experimental group. The scores of emotion perception **(A)**, emotional response acceptance **(B)**, emotion understanding **(C)**, goal-directed behavior stimulation **(D)**, effective use of regulatory strategies **(E)**, and emotional impulse control **(F)**. ^*^p < 0.05 and ^***^p < 0.001.

For experimental group with GIMT intervention, the scores of emotional response acceptance (Figure 5B), emotional understanding (Figure 5C), goal-directed behavior stimulation (Figure 5D), effective use of regulatory strategies (Figure 5E), and emotional impulse control (Figure 5F) were significantly lower than those before the intervention. However, the score of emotional perception was upregulated (Figure 5A). Excluding the evaluation of emotional perception, the emotional regulation ability of the experimental group was significantly improved.

Statistics by gender showed that in the control group, the emotional regulation and impulse control of boys and girls were all decreased significantly, but there was no gender difference (Tables 4, 5). In the experimental group, the improvement of emotional regulation in girls was significantly higher than that in boys (Table 4), especially in the emotional impulse control dimension (Table 5).

**Table 4:** Gender differences of GIMT effect in emotional regulation.

Emotional regulation	Gender	Before	After	*p*
Control group	Male	116.25 ± 10.69	125.75 ± 7.37	0.0103^*^
	Female	121 ± 9.62	129.07 ± 8.13	0.0167^*^
Experimental group	Male	118.4 ± 14.62	103.79 ± 12.78	0.0042^*^
	Female	116.7 ± 14.43	100.5 ± 7.08	<0.0001^***^

* *p < 0.05 and*

*** *p < 0.001*.

**Table 5:** Gender differences of GIMT effect in emotional impulse control.

Emotional impulse control	Gender	Before	After	*p*
Control group	Male	19.13 ± 2.31	21.31 ± 2.02	0.0121^*^
	Female	19.43 ± 2.28	21.07 ± 2.27	0.0451^*^
Experimental group	Male	18.8 ± 3.12	15.57 ± 3.39	0.0070^*^
	Female	18.19 ± 3.12	14 ± 2.41	<0.0001^**^

* *p < 0.05 and*

** *p < 0.001*.

### Follow-up Analyses the Qualitative Reports of Subjects’ Emotions

The follow-up analyses showed that the students in the experimental group showed different degrees of improvement in emotional regulation after GIMT. The specific improvements were reflected in the following aspects: (a) They could better accept and talk about themselves and their emotional experience, no matter the experience is positive or negative; (b) The goal behavior ability, decision-making ability, and execution ability were improved, the subjective initiative in dealing with negative emotions was stronger, and the task could be completed better; (c) Their impulse control ability had been improved, and they could take the initiative to deal with negative emotions to make themselves feel better, such as listening to music, sports, or communicating with friends with a more open attitude; (d) They were more open to the environment, more willing to listen to others and participate in collective activities; (e) They had improved their ability to cope with pressure, make choices, and have more confidence in themselves; (f) Their emotional awareness became clearer and began to learn to understand and accept their negative feelings. Some students even felt happy with their changes; (g) They had stronger ability of emotional regulation and could better deal with the relationship with themselves, others, study or work; (h) They could reassess their situation more actively, such as reducing unnecessary worry or anxiety, looking at problems from a positive or pluralistic perspective and thinking logically; and (i) No students showed a decline in emotional regulation.

Meanwhile, all students in the experimental group showed improvement in depression: (a) They no longer felt so sad, nervous, or anxious; (b) They could experience more happiness and interests such as music, sports, learning, and reading, and are more willing to be with friends and family; (c) Their appetite and sleep quality were improved; (e) Their self-identity and sense of value increased, and their sense of self-blame or self-guilt decreased; (f) They were more energetic and their focus on study and work was improved; (g) They were more optimistic about the future and lived a fulfilling life; (h) They no longer had repeated thoughts or ideas about death; and (i) No students showed increased levels of depression.

The follow-up of the control group showed that there were no positive changes in the improvement of emotional regulation and depressive symptoms.

## Discussion

Our research showed that excluding the “emotional perception” dimension of DERS, the emotional regulation ability of the experimental group was significantly improved after GIMT intervention and that of the control group was significantly decreased. Both quantitative analyses and qualitative information supported this result, which also laterally verified the research conclusions of Park et al. (2012). It is worth mentioning that after GIMT intervention, the alteration trend of “emotional perception” in the two groups was opposite to the other five dimensions. A study of Norwegian showed that except for “emotional perception,” the other five dimensions showed chronbach’s alpha >0.91 (Endre et al., 2017). This finding was consistent with other studies (Osborne and Michonsk, 2017; Hallion et al., 2018), which prove that it is reasonable to exclude the “emotional perception” dimension when using DERS (Mbna et al., 2020). After GIMT intervention, the level of depression in the experimental group decreased significantly, but there was no significant difference in the control group.

The follow-up of 4 weeks after the intervention showed that the students in the experimental group were easier to accept and deal with negative emotions. They had stronger impulse control ability and established more extensive emotional regulation strategies, such as listening to music, taking exercise, and other healthy ways. Their moods were more stable, their senses of pressure were reduced, and their anxiety was significantly relieved. They experienced more happiness. Their curiosity and interest levels were increased. They were more satisfied with the reality and more optimistic about the future. Some people no longer had suicidal thoughts or ideas.

The improvement of emotional regulation and depression in the experimental group may be related to the improvement of impulse control ability. Annamarie et al. found that enhancing impulse control ability was a key factor in the treatment of depression (Defayette et al., 2021). Our findings also indicated that GIMT may be more suitable for the emotional expression characteristics of girls as the improvement of impulse control, total emotional regulation, and depression of girls were significantly higher than those of boys in the experimental group.

In terms of emotional improvement, GIMT uses improvisation music therapy based on Priestleys’ psychodynamic orientation (Priestley, 1994). Through improvisation and language discussion, participants can be encouraged to accept themselves in music interaction and promote the solution of emotional and cognitive problems. In addition, mirroring, dialogue, and synchronization technologies can help to evoke the experience and expression of emotional significance (Bruscia, 1987), so as to achieve the overall improvement of emotion regulation and depression (Aalbers et al., 2020). The research of Erkkila et al. (2008, 2011) also proved this conclusion.

Moreover, as a nonverbal social communication activity, GIMT applies the principle of free improvisation in behavior improvement (Bruscia, 1987). GIMT encourages participants to improvise music to promote emotional expression and interpersonal communication, enhance social skills, elevate self-esteem and self-confidence, and create conditions for the optimization of interpersonal environment. This was also verified in the study of Porter et al. (2017).

This paper explored the intervention effect of GIMT on emotional regulation and depression for the first time. The changes in the experimental group supported the hypothesis that GIMT might enhance emotional regulation and improve depression symptoms, consistent with the research results of Fachner et al. (2013). They found that impromptu music therapy directly affected the cerebral cortex activity of adult patients with depression. The therapy induced frontotemporal nerve reorganization and then improved depressive symptoms (Fachner et al., 2013). The research of Aalbers et al. validated the above hypothesis from the perspective of individual treatment and supported the internal mechanism of GIMT to improve depression by improving emotional regulation (Aalbers et al., 2019, 2020).

Impromptu concert can affect the brain function of patients so as to alleviate and improve emotional problems. Impromptu concert can activate amygdala (Holland and Gallagher, 2004; Roozendaal et al., 2009), hippocampus (Chanda and Levitin, 2013; Koelsch, 2014), nucleus accumbens (Blood and Zatorre, 2001), insula (Pessoa et al., 2002; Trost et al., 2015), thalamus (Pessoa et al., 2002; Trost et al., 2015), and anterior cingulate cortex (Brown et al., 2004; de Manzano and Ullen, 2012; Moore, 2013), and promote dopamine secretion (Menon and Levitin, 2005). These changes can reduce stress, enhance positive emotional experience, improve impulse control ability, and finally improve emotional regulation and depressive symptoms. These findings also explain the conclusions of Annamarie et al. from a physiological level (Defayette et al., 2021).

GIMT uses a variety of percussion instruments with multiple pitches and rich timbres. The esthetic experience of impromptu music can reduce the secretion of adrenaline and noradrenaline, promote the secretion of endorphins by the pituitary gland, and stimulate the peak experience of players. Maslow (1998) found that peak experience could make positive changes in self-evaluation and promote individuals to solve problems more actively and creatively, which is the “source power” to enhance emotional regulation and promote the improvement of depression.

## Limitation

There are also some limitations in our study. Firstly, only college students from one university were included. Although the sample size is enough to explore and verify the effect of GIMT, further research is still needed on similar groups and other different groups to provide evidence for the universality of its effect. Secondly, the follow-up time of this study was extended to 4 weeks after GIMT. But we are not sure how long the positive impact of GIMT will last. Third, the study leader has an unavoidable dual role—researcher and music therapist, which may lead to bias and overestimation of the effect. Fourth, the qualitative conclusion is based on semi-structured interview. It is difficult for researchers to avoid subjective tendency. If the structured and standardized qualitative data collection method are adopted, the objectivity can be improved. In general, solving these limitations are necessary for the more accurate understanding of the function of GIMT.

## Conclusion

GIMT, as an intervention program, plays an important and effective role in improving the emotional regulation ability of college students and reducing depressive symptoms.

## Data Availability Statement

The original contributions presented in the study are included in the article/supplementary material, further inquiries can be directed to the corresponding authors.

## Ethics Statement

The studies involving human participants were reviewed and approved by ethics committee of the Affiliated Hospital of Qingdao University. The patients/participants provided their written informed consent to participate in this study.

## Author Contributions

LZ and WY designed the study. MZ carried out the GIMT experiments and drafted the manuscript. YD, JZ, and XJ assisted in the recruitment and screening of experimental subjects. LZ and NX analyzed the data and made the graphics. All authors contributed to the article and approved the submitted version.

## Funding

This study was supported by the MOE (Ministry of Education in China) Project of Humanities and Social Sciences (Grant No: 20JDSZ3122).

## Conflict of Interest

The authors declare that the research was conducted in the absence of any commercial or financial relationships that could be construed as a potential conflict of interest.

## Publisher’s Note

All claims expressed in this article are solely those of the authors and do not necessarily represent those of their affiliated organizations, or those of the publisher, the editors and the reviewers. Any product that may be evaluated in this article, or claim that may be made by its manufacturer, is not guaranteed or endorsed by the publisher.

## References

[ref1] Aalbers S. Fusar-Poli L. Freeman R. E. Spreen M. Ket J. C. F. Vink A. C.. (2017). Music therapy for depression. Cochrane Database Syst. Rev. 2017:CD004517. doi: 10.1002/14651858.CD004517.pub3, PMID: 29144545 PMC6486188

[ref2] Aalbers S. Spreen M. Pattiselanno K. Verboon P. Vink A. van Hooren S. (2020). Efficacy of emotion-regulating improvisational music therapy to reduce depressive symptoms in young adult students: a multiple-case study design. Arts Psychother. 71:101720. doi: 10.1016/j.aip.2020.101720

[ref3] Aalbers S. Vink A. Freeman R. E. Pattiselanno K. Spreen M. van Hooren S. (2019). Development of an improvisational music therapy intervention for young adults with depressive symptoms: An intervention mapping study. Arts Psychother. 65:101584. doi: 10.1016/j.aip.2019.101584

[ref4] Backer J. D. (2008). Music and psychosis. Nord. J. Music. Ther. 17, 89–104. doi: 10.1080/08098130809478202

[ref5] Barker M. M. Beresford B. Bland M. Fraser L. K. (2019). Prevalence and incidence of anxiety and depression Among children, adolescents, and young adults With life-limiting conditions: a systematic review and meta-analysis. JAMA Pediatr. 173, 835–844. doi: 10.1001/jamapediatrics.2019.1712, PMID: 31282938 PMC6618774

[ref6] Berking M. Wirtz C. M. Svaldi J. Hofmann S. G. (2014). Emotion regulation predicts symptoms of depression over five years. Behav. Res. Ther. 57, 13–20. doi: 10.1016/j.brat.2014.03.003, PMID: 24754907

[ref7] Blood A. J. Zatorre R. J. (2001). Intensely pleasurable responses to music correlate with activity in brain regions implicated in reward and emotion. Proc. Natl. Acad. Sci. 98, 11818–11823. doi: 10.1073/pnas.191355898, PMID: 11573015 PMC58814

[ref8] Boyd R. C. Le H. N. Somberg R. (2005). Review of screening instruments for postpartum depression. Arch. Womens Mental Health 8, 141–153. doi: 10.1007/s00737-005-0096-6 16133785

[ref9] Breedvelt J. J. F. Kandola A. Kousoulis A. A. Brouwer M. E. Karyotaki E. Bockting C. L. H.. (2018). What are the effects of preventative interventions on major depressive disorder (MDD) in young adults? A systematic review and meta-analysis of randomized controlled trials. J. Affect. Disord. 239, 18–29. doi: 10.1016/j.jad.2018.05.010, PMID: 29990660

[ref10] Brown S. Martinez M. J. Parsons L. M. (2004). Passive music listening spontaneously engages limbic and paralimbic systems. Neuroreport 15, 2033–2037. doi: 10.1097/00001756-200409150-00008, PMID: 15486477

[ref11] Bruscia K. (1987). Improvisational Models of Music Therapy. Springfield, IL: Charles C Thomas Publisher.

[ref12] Chanda M. L. Levitin D. J. (2013). The neurochemistry of music. Trends Cogn. Sci. 17, 179–193. doi: 10.1016/j.tics.2013.02.007, PMID: 23541122

[ref13] Compare A. Zarbo C. Shonin E. Gordon W. V. Marconi C. (2014). Emotional regulation and depression: a potential mediator between heart and mind. Cardiovasc. Psychiatry Neurol. 2014:324374, 1–10. doi: 10.1155/2014/324374, PMID: 25050177 PMC4090567

[ref14] Conley C. S. Shapiro J. B. Kirsch A. C. Durlak J. A. (2017). A meta-analysis of indicated mental health prevention programs for at-risk higher education students. J. Couns. Psychol. 64, 121–140. doi: 10.1037/cou0000190, PMID: 28277730

[ref15] de Manzano O. Ullen F. (2012). Goal-independent mechanisms for free response generation: creative and pseudo-random performance share neural substrates. NeuroImage 59, 772–780. doi: 10.1016/j.neuroimage.2011.07.016, PMID: 21782960

[ref16] Defayette A. B. Whitmyre E. D. Lopez R. Brown B. Wolff J. C. Spirito A.. (2021). Adolescent depressed mood and difficulties with emotion regulation: concurrent trajectories of change. J. Adolesc. 91, 1–14. doi: 10.1016/j.adolescence.2021.07.001, PMID: 34252783 PMC8380718

[ref17] Endre V. Lin S. Berge O. Svendsen J. L. Per-Einar B. Elisabeth S. (2017). The association between self-reported difficulties in emotion regulation and heart rate variability: The salient role of not accepting negative emotions. Front. Psychol. 8:328. doi: 10.3389/fpsyg.2017.00328 28337160 PMC5343522

[ref18] Erkkila J. Brabant O. Saarikallio S. Ala-Ruona E. Hartmann M. Letule N.. (2019). Enhancing the efficacy of integrative improvisational music therapy in the treatment of depression: study protocol for a randomised controlled trial. Trials 20:244. doi: 10.1186/s13063-019-3323-6, PMID: 31036058 PMC6489303

[ref19] Erkkila J. Gold C. Fachner J. Ala-Ruona E. Punkanen M. Vanhala M. (2008). The effect of improvisational music therapy on the treatment of depression: protocol for a randomised controlled trial. BMC Psychiatry 8:50. doi: 10.1186/1471-244x-8-50, PMID: 18588701 PMC2474861

[ref20] Erkkila J. Punkanen M. Fachner J. Ala-Ruona E. Pontio I. Tervaniemi M.. (2011). Individual music therapy for depression: randomised controlled trial. Br. J. Psychiatry 199, 132–139. doi: 10.1192/bjp.bp.110.085431 21474494

[ref21] Fachner J. Gold C. Erkkila J. (2013). Music therapy modulates Fronto-temporal activity in rest-EEG in depressed clients. Brain Topogr. 26, 338–354. doi: 10.1007/s10548-012-0254-x, PMID: 22983820

[ref22] Fetters M. D. Curry L. A. Creswell J. W. (2013). Achieving integration in mixed methods designs-principles and practices. Health Serv. Res. 48, 2134–2156. doi: 10.1111/1475-6773.12117, PMID: 24279835 PMC4097839

[ref23] Fetters M. D. Molina-Azorin J. F. (2019). In This issue: innovations in mixed methods—causality, case study research With a circular joint display, social media, grounded theory, and phenomenology. J. Mixed Methods Res. 13, 123–126. doi: 10.1177/1558689819834986

[ref24] Folkman S. Moskowitz J. T. (2004). Coping: pitfalls and promise. Annu. Rev. Psychol. 55, 745–774. doi: 10.1146/annurev.psych.55.090902.141456, PMID: 14744233

[ref25] Gratz K. L. Roemer L. (2004). Multidimensional assessment of emotion regulation and dysregulation: development, factor structure, and initial validation of the difficulties in emotion regulation scale. J. Psychopathol. Behav. Assess. 26, 41–54. doi: 10.1023/B:Joba.0000007455.08539.94

[ref26] Gross J. J. (2002). Emotion regulation: affective, cognitive, and social consequences. Psychophysiology 39, 281–291. doi: 10.1017/S0048577201393198, PMID: 12212647

[ref27] Hallion L. S. Steinman S. A. Tolin D. F. Diefenbach G. J. (2018). Psychometric properties of the difficulties in emotion regulation scale (DERS) and its short forms in adults with emotional disorders. Front. Psychol. 9:539. doi: 10.3389/fpsyg.2018.00539, PMID: 29725312 PMC5917244

[ref28] Herwig U. Opialla S. Cattapan K. Wetter T. C. Jancke L. Bruhl A. B. (2018). Emotion introspection and regulation in depression. Psy. Res. Neuro. 277, 7–13. doi: 10.1016/j.pscychresns.2018.04.008, PMID: 29778804

[ref29] Holahan C. J. Moos R. H. (1986). Personality, coping, and family resources in stress resistance: a longitudinal analysis. J. Pers. Soc. Psychol. 51, 389–395. doi: 10.1037//0022-3514.51.2.389, PMID: 3746619

[ref30] Holland P. C. Gallagher M. (2004). Amygdala-frontal interactions and reward expectancy. Curr. Opin. Neurobiol. 14, 148–155. doi: 10.1016/j.conb.2004.03.007, PMID: 15082318

[ref31] Hou J. C. Song B. Chen A. C. N. Sun C. G. Zhou J. X. Zhu H. D.. (2017). Review on neural correlates of emotion regulation and music: implications for emotion Dysregulation. Front. Psychol. 8:501. doi: 10.3389/fpsyg.2017.00501, PMID: 28421017 PMC5376620

[ref32] Ibrahim A. K. Kelly S. J. Adams C. Glazebrook C. (2013). A systematic review of studies of depression prevalence in university students. J. Psychiatr. Res. 47, 391–400. doi: 10.1016/j.jpsychires.2012.11.015 23260171

[ref33] Jaakko E. (2004). From signs to symbols, from symbols to words. Voices A World Forum for Music Therapy 4. doi: 10.15845/voices.v4i2.176

[ref34] Joormann J. Quinn M. E. (2014). Cognitive processes and emotion regulation in depression. Depress. Anxiety 31, 308–315. doi: 10.1002/da.22264 24668779

[ref35] Joormann J. Stanton C. H. (2016). Examining emotion regulation in depression: A review and future directions. Behav. Res. Ther. 86, 35–49. doi: 10.1016/j.brat.2016.07.007, PMID: 27492851

[ref36] Koelsch S. (2014). Brain correlates of music-evoked emotions. Nat. Rev. Neurosci. 15, 170–180. doi: 10.1038/nrn3666, PMID: 24552785

[ref37] Koelsch S. Skouras S. Fritz T. Herrera P. Bonhage C. Kussner M. B.. (2013a). The roles of superficial amygdala and auditory cortex in music-evoked fear and joy. NeuroImage 81, 49–60. doi: 10.1016/j.neuroimage.2013.05.008, PMID: 23684870

[ref38] Koelsch S. Skouras S. Jentschke S. (2013b). Neural correlates of emotional personality: a structural and functional magnetic resonance imaging study. PLoS One 8:e77196. doi: 10.1371/journal.pone.0077196, PMID: 24312166 PMC3842312

[ref39] Macdonald H. Z. Price J. L. (2018). The role of emotion regulation in the relationship between empathy and internalizing symptoms in college students. Mental health. Prevention 13, 43–49. doi: 10.1016/j.mhp.2018.11.004

[ref40] Manian N. Schmidt E. Bornstein M. H. Martinez P. (2013). Factor structure and clinical utility of BDI-II factor scores in postpartum women. J. Affect. Disord. 149, 259–268. doi: 10.1016/j.jad.2013.01.039, PMID: 23521870 PMC3672272

[ref41] Maratos A. Crawford M. J. Procter S. (2011). Music therapy for depression: it seems to work, but how? Br. J. Psychiatry 199, 92–93. doi: 10.1192/bjp.bp.110.087494, PMID: 21804144

[ref42] Marik M. Stegemann T. (2016). Introducing a new model of emotion dysregulation with implications for everyday use of music and music therapy. Music. Sci. 20, 53–67. doi: 10.1177/1029864915622055

[ref43] Maslow A. H. (1998). Toward a Psychology of Being, 3rd *Edn*. United States: John Wiley & Sons Inc.

[ref44] Mausbach B. T. Moore R. Roesch S. Cardenas V. Patterson T. L. (2010). The relationship Between homework compliance and therapy outcomes: An updated meta-analysis. Cogn. Ther. Res. 34, 429–438. doi: 10.1007/s10608-010-9297-z, PMID: 20930925 PMC2939342

[ref45] Mbna B. Jhba C. Rwa B. Tpa B. (2020). Does cognitive behavioural therapy or mindfulness-based therapy improve mental health and emotion regulation among men who perpetrate intimate partner violence? A randomised controlled trial - science direct. Int. J. Nurs. Stud. 113:103795, doi: 10.1016/j.ijnurstu.2020.103795 33120137

[ref46] Menon V. Levitin D. J. (2005). The rewards of music listening: response and physiological connectivity of the mesolimbic system. NeuroImage 28, 175–184. doi: 10.1016/j.neuroimage.2005.05.053, PMID: 16023376

[ref47] Moore K. S. (2013). A systematic review on the neural effects of music on emotion regulation: implications for music therapy practice. J. Music. Ther. 50, 198–242. doi: 10.1093/jmt/50.3.198, PMID: 24568004

[ref48] Nolen-Hoeksema S. (2012). Emotion regulation and psychopathology: the role of gender. Annu. Rev. Clin. Psychol. 8, 161–187. doi: 10.1146/annurev-clinpsy-032511-143109 22035243

[ref49] Osborne T. L. Michonsk J. (2017). Factor structure of the difficulties in emotion regulation scale (DERS) in adult outpatients receiving dialectical behavior therapy (DBT). J. Psychopathol. Behav. Assess. 39, 355–371. doi: 10.1007/s10862-017-9586-x

[ref50] Park C. L. Edmondson D. Lee J. (2012). Development of self-regulation abilities as predictors of psychological adjustment Across the first year of college. J. Adult Dev. 19, 40–49. doi: 10.1007/s10804-011-9133-z

[ref51] Pessoa L. McKenna M. Gutierrez E. Ungerleider L. G. (2002). Neural processing of emotional faces requires attention. Proc. Natl. Acad. Sci. U. S. A. 99, 11458–11463. doi: 10.1073/pnas.172403899, PMID: 12177449 PMC123278

[ref52] Porter S. McConnell T. McLaughlin K. Lynn F. Cardwell C. Braiden H. J.. (2017). Music therapy for children and adolescents with behavioural and emotional problems: a randomised controlled trial. J. Child Psychol. Psychiatry 58, 586–594. doi: 10.1111/jcpp.12656, PMID: 27786359

[ref53] Priestley M. (1994). Essays on Analytical Music Therapy. New Braunfels: Barcelona Publishers.

[ref54] Roozendaal B. McEwen B. S. Chattarji S. (2009). Stress, memory and the amygdala. Nat. Rev. Neurosci. 10, 423–433. doi: 10.1038/nrn2651 19469026

[ref55] Rotter J. B. Chance J. E. Phares E. J. (1972). Applications of a social learning theory of personality. *>Gale encyclopedia of psychology edition*. 574–608.

[ref56] Surmann A. T. (1999). Negative mood regulation expectancies, coping, and depressive symptoms Among American nurses. J. Soc. Psychol. 139, 540–543. doi: 10.1080/00224549909598415, PMID: 10457766

[ref57] Trost W. Fruhholz S. Cochrane T. Cojan Y. Vuilleumier P. (2015). Temporal dynamics of musical emotions examined through intersubject synchrony of brain activity. Soc. Cogn. Affect. Neurosci. 10, 1705–1721. doi: 10.1093/scan/nsv060, PMID: 25994970 PMC4666110

[ref58] Vos T. Barber R. M. Bell B. Bertozzi-Villa A. Biryukov S. Bolliger I.. (2015). Global, regional, and national incidence, prevalence, and years lived with disability for 301 acute and chronic diseases and injuries in 188 countries, 1990-2013: a systematic analysis for the global burden of disease study 2013. Lancet 386, 743–800. doi: 10.1016/S0140-6736(15)60692-4, PMID: 26063472 PMC4561509

[ref59] Wigram T. (2004). Improvisation: Methods and Techniques for Music Therapy Clinicians, Educators and Students. London, England: Jessica Kingsley Publishers.

[ref60] Wu P. C. Huang T. W. (2014). Gender-related invariance of the Beck depression inventory II for Taiwanese adolescent samples. Assessment 21, 218–226. doi: 10.1177/1073191112441243, PMID: 22517921

